# Hospital Saturday Fund

**Published:** 1909-04-24

**Authors:** 


### HOSPITAL SATURDAY FUND.

Sir Savile Crossley presided on Saturday last, April 17, at the annual meeting of the supporters of the Hospital Saturday Fund, at the Mansion House. The income last year was £29,830—£2,690 more than in the previous year. The amount collected by the local committees exceed £4,000, as compared with £3,529 in 1907. £27,332 was distributed among 209 institutions. About 6,000 penny-a-week sheets were issued quarter by quarter, and 4,500 annual sheets were distributed in June. The Surgical Appliance Committee supplied 5,318 appliances during the year at a cost of £2,387, towards which the recipients or their friends contributed £1,252. The Hon. Mrs. John Coke, the daughter of their treasurer (the Hon. Harry Lawson), distributed the medallions of the St. John Ambulance Association. In 1896 the total invested capital of the Central Funds was nil, but now in 1909 the three great funds have an invested capital worth well over one and a half million. In 1896 the amount distributed by the Central Funds was £60,000, while last year it had risen to £238,000. The starting of the King's Fund so far from damaging the hospitals had very materially benefited them. Canon J. W. Horsley moved the adoption of the report, and the motion was seconded by the Mayor of Finsbury, supported by the Mayor of Woolwich, and adopted unanimously.

Dr. Acland pointed out that one-sixth of the fund was devoted to the crusade against tuberculosis, which was the cause of the death of 100,000 people a year in England alone. Tuberculosis was far more prevalent than it was supposed to be, and he advocated the notification of the disease and its more general treatment.

